# Adipose-Derived Stromal Cells Seeded on Integra^®^ Dermal Regeneration Template Improve Post-Burn Wound Reconstruction

**DOI:** 10.3390/bioengineering7030067

**Published:** 2020-07-02

**Authors:** Marcin Piejko, Karolina Radziun, Sylwia Bobis-Wozowicz, Agnieszka Waligórska, Eliza Zimoląg, Michał Nessler, Anna Chrapusta, Zbigniew Madeja, Justyna Drukała

**Affiliations:** 1 Cell Bank, Department of Cell Biology, Faculty of Biochemistry, Biophysics and Biotechnology, Jagiellonian University, 30-387 Krakow, Poland; marcin.piejko@uj.edu.pl (M.P.); radziun.k@gmail.com (K.R.); sylwia.bobis@uj.edu.pl (S.B.-W.); eliza.laczna@gmail.com (E.Z.); z.madeja@uj.edu.pl (Z.M.); 2 Department of General Surgery, Jagiellonian University Medical College, 31-215 Krakow, Poland; 3 Department of Cell Biophysics, Faculty of Biochemistry, Biophysics and Biotechnology, Jagiellonian University, 30-387 Krakow, Poland; agnieszka.waligorska@uj.edu.pl; 4 Malopolska Center for Burns and Plastic Surgery, The Ludwik Rydygier Hospital, 31-826 Krakow, Poland; mnessler@gmail.com (M.N.); anna.chrapusta@gmail.com (A.C.)

**Keywords:** cell-based therapy, collagen-based matrix, tissue engineering, burn-related scars, wound healing

## Abstract

Fibrosis of burn-related wounds remains an unresolved clinical issue that leads to patient disability. The aim of this study was to assess the efficacy of the transplantation of adipose-derived stromal cells seeded onto a collagen-based matrix in the reconstruction of burn-related scars. Here, we characterized an in vitro interaction between adipose-derived stromal cells and a collagen-based matrix, Integra^®^DRT. Our results show that transcription of pro-angiogenic, remodeling, and immunomodulatory factors was more significant in adipose-derived stromal cells than in fibroblasts. Transcription of metalloproteinases 2 and 9 is positively correlated with the collagenolytic activity of the adipose-derived stromal cells seeded onto Integra^®^DRT. The increase in the enzymatic activity corresponds to the decrease in the elasticity of the whole construct. Finally, we validated the treatment of a post-excision wound using adipose-derived stromal cells and an Integra^®^DRT construct in a 25-year-old woman suffering from burn-related scars. Scarless healing was observed in the area treated by adipose-derived stromal cells and the Integra^®^DRT construct but not in the reference area where Integra^®^DRT was applied without cells. This clinical observation may be explained by in vitro findings: Enhanced transcription of the vascular endothelial growth factor as well as remodeling of the collagen-based matrix decreased mechanical stress. Our experimental treatment demonstrated that the adipose-derived stromal cells seeded onto Integra^®^DRT exhibit valuable properties that may improve post-excision wound healing and facilitate skin regeneration without scars.

## 1. Introduction

Skin and subcutaneous tissue provide easily accessible sources of regenerative cells such as keratinocytes [1], human skin fibroblasts [2], and adipose-derived stromal cells (ASCs) [3]. Whereas, keratinocyte-based therapies have advanced from an experimental to standard treatment of wounds [1], ASCs are considered in numerous medical indications, from the regeneration of perianal fistula and facilitating wound healing to bone reconstruction [2,4,5,6]. Adipose-derived stem cells, in addition to their structural functions, maintain capacity for soft tissue regeneration as well as pro-angiogenic, pro-migratory, and immunomodulatory activities [7]. Destruction of the dermis in third- and fourth-degree burns as well as factors affecting wound healing, such as infections, interfere with primary wound healing providing fibrosis. Joint contractures caused by scars lead to disability and necessitate the use of reconstructive surgery [8,9], which is a viable option only when healthy skin is accessible for auto transplantation. As a consequence, for large post-burn scars, collagen-based dermal substitutes such as Integra^®^DRT [10,11], AlloDerm [12], and Acellular Dermal Matrix [9] are combined with surgical treatment to manage the post-excision area.

Integra^®^DRT consists of bovine collagen I crosslinked with chondroitin sulfates harvested from sharks, creating three-dimensional scaffolds providing both a material for post-excision dressing and a biocompatible matrix for vascularization and remodeling.

Typically, for surgical reconstruction of a post-excision wound, the dermal template is assumed to be vascularized spontaneously from the wound bed [13]. Insufficient angiogenesis increases the risk of infections caused by the contaminating bacteria that grow in the biomaterial volume inaccessible to immune cells [14]. Supposedly, fibroblasts migrating from the wound bed and penetrating Integra^®^DRT should be exposed to mechanical stress at the border between the host tissue and the biomaterial. The mechanical stress is a well-known and strong profibrotic factor [15,16] that may produce a scar at the border between Integra^®^DRT and healthy skin. Taken together, prolonged vascularization, infections, and scar formation around the biomaterial sheet are the clinical issues that negatively impact therapeutic efforts.

ASCs provide features that appear to be a complementary solution to wound healing challenges. These clinically useful cells have been widely explored in numerous attempts at regeneration of soft [4,5] and hard [17] tissues. ASCs respond to microenvironmental conditions by Vascular endothelial growth factor (VEGF) expression [18], immunomodulation [7], stimulation of fibroblasts migration [18], or by modulation of tissue remodeling (by maintaining the balance between metalloproteinases (MMPs) and tissue inhibitors of metalloproteinases (TIMPs) [19] and others). A series of clinical trials that tested auto- and allogenic sources of ASCs demonstrated that enhancing soft tissue healing is independent of the auto- or allogenic sources of these cells. Liu et al. revealed the anti-fibrotic effect of ASCs on fibroblasts as being more related to ASC secretome than to direct cell–cell interaction [20].

The aim of this study was to explain the rationale behind the use of ASCs in combination with a clinically approved collagen-based scaffold for the reconstruction of burn-related scars. Here, we characterized an in vitro interaction between adipose-derived stromal cells and collagen-based matrix, Integra^®^DRT. Finally, we validated the concept of ASCs and the Integra^®^DRT construct in a case-based study of burn-related scar reconstruction.

## 2. Materials and Methods

### 2.1. ASCs and Human Skin Fibroblasts Isolation and Culture

The human adipose-derived stromal cells and human skin fibroblasts (HSFs) were harvested from full thickness skin and subcutaneous adipose tissue from healthy donors qualified for abdominoplasty. Tissue samples were obtained in accordance with the positive opinion of the Jagiellonian University—Medical Collage Bioethical Board (1072.6120.10.2017). In brief, around 3 mL of adipose tissue was separated from the subcutaneous layer of skin, washed three times with phosphate buffered saline (PBS) supplemented with 100 U/mL penicillin and 0.1 mg/mL streptomycin (P/S) (all chemicals obtained from Sigma-Aldrich, St. Louis, MO, USA), and digested with 1 mg/mL collagenase I from *Clostridium histolyticum* (Sigma-Aldrich, St. Louis, MO, USA) at 37 °C for 60 min with shaking several times. Then, the mixture was diluted twice in low glucose Dulbecco’s modified essential medium (DMEM LG; Sigma-Aldrich, St. Louis, MO, USA) supplemented with 10% fetal bovine serum (FBS; Gibco, Darmstadt, Germany), P/S, and centrifuged at 200× *g* for 10 min. The stromal vascular fraction was resuspended in a culture medium and plated onto a T-25 flask. Erythrocytes were washed out by replacement of the medium. During cell culture, the medium was replaced every 3 days and ASCs were expanded up to 70% confluency and passages were performed with 0.05% trypsin with 2 mmol/L EDTA solution (Sigma-Aldrich, St. Louis, MO, USA).

HSFs were isolated from the dermis after epidermis detachment followed by digestion with 5 U/mL dispase (Stem Cells Technologies, Cologne, Germany). The dermis was cut into small pieces and digested in solution composed of DMEM LG supplemented with 10% FBS, P/S, and 1 mg/mL collagenase I (Sigma-Aldrich, St. Louis, MO, USA). After 24 h incubation at 37 °C, supernatant from the non-digested dermis was collected to a fresh tube and centrifuged at 200× *g* for 10 min at room temperature. The pellet was resuspended in high glucose DMEM (DMEM HG; Sigma-Aldrich, St. Louis, MO, USA) supplemented with 10% FBS and P/S. The medium was replaced every 3 days. Cells were cultured up to 80% confluency and passages were performed by using 0.05% trypsin with 2 mmol/L EDTA solution.

### 2.2. Preparation of ASC–Integra^®^DRT Constructs

The cell–biomaterial constructs were prepared by cutting Integra^®^DRT (Integra Sciences, Plainsboro Township, NJ, USA) with a 6 mm diameter puncher (Premier Uni-Punch, Plymouth Meeting, PA, USA), the same as the diameter of wells in 96-well plates. Tightly fitted roundels were placed inside a 96-well plate and washed three times with sterile PBS. Finally, the constructs were pre-incubated in the cell culture medium overnight. Then, the medium was replaced and 2 × 10^4^ ASCs were transferred into each well.

### 2.3. Growth Assay

Cells growth was assessed using a water-soluble tetrazolium salt 1 (WST-1; Sigma-Aldrich, St. Louis, MO, USA) assay on the next day, and 1, 2, and 3 weeks after cell seeding. We seeded 2 × 10^4^ ASCs from four donors in duplicates on the top of the biomaterial. After overnight incubation, biomaterial–cell constructs were transferred to a new 96-well plate. The WST-1 assay was performed according to the manufacturer’s instructions. In brief, the medium was replaced with a fresh one supplemented with 10% WST-1 (*v*/*v*). Cells were incubated at 37 °C in 5% CO_2_ for up to 3 h. After this time, the medium was transferred to a new 96-well plate and absorbance at 490 and 605 nm was measured in Multiskan™ FC Microplate Photometer (Thermo Fisher Scientific, Waltham, MA, USA). The biomaterial–cell constructs were washed three times with a fresh cell culture medium and were maintained at 37 °C, 5% CO_2_ until the next measurement. Cell culture media were replaced twice per week.

### 2.4. Confocal Microscopy

Cells adhering to Integra^®^DRT were imaged with a Leica TCS SP5 II confocal laser scanning microscope (Leica Microsystems, Mannheim, Germany) equipped with argon and HeNe lasers. Tetramethylrhodamine, ethyl ester (TMRE; Thermo Fisher Scientific, Waltham, MA, USA) was used to visualize living cells. As a cell-permeable and cationic dye, TMRE concentrates on negatively charged mitochondria. After 3 weeks of culturing of ASCs onto Integra^®^DRT, the constructs were washed three times with a fresh medium with no phenol red. Solutions of Hoechst 33342 (Thermo Fisher Scientific, Waltham, MA, USA) and TMRE were pipetted directly to the medium to the final concentrations of 2.2 and 0.1 µM, respectively, and subsequently the constructs were incubated at 37 °C and 5% CO_2_. After 5 min, an objective PL APO 40.0 × 1.25 with oil immersion was applied to capture 3D images. Lasers at 405, 488, and 543 nm were used for excitation. Hoechst emission was recorded with sequential scanning option within 415–465 nm, TMRE in the range of 570 to 695 nm, and Integra^®^DRT autofluorescence in the range of 500–535 nm. The confocal pinhole size was set to 1 Airy unit. During the imaging process, samples were maintained at 37 °C. The obtained 3D images were analyzed using ImageJ-Fiji Software [21]. The long-range Integra autofluorescence necessitated the removal of this signal from the rest of channels, which was achieved by subtraction of the green channel from the blue and red channels.

### 2.5. Proteolytic Activity of ASC–Integra^®^DRT Constructs

The collagenolytic activity of ASC–Integra^®^DRT constructs were assessed using an EnzCheck collagenase activity kit (Thermo Fisher Scientific, Waltham, MA, USA) according to the manufacturer’s protocol. ASC–Integra^®^DRT constructs were prepared similarly to the growth assay in a 96-well plate. On the 2nd, 7th, and 14th day after cell seeding, the medium was replaced with 200 µL of 20 µg/mL EnzCheck substrate and incubated for 60 min at 37 °C in a cell culture incubator. Then, the liquid was transferred to a 96-well black plate and fluorescence was measured using a Synergy H1 microplate reader (excitation: 485 nm, emission: 528 nm; BioTek, Winooski, VT, USA). All data were normalized against the signal obtained for the negative (EnzCheck substrate) and the positive (EnzCheck substrate supplemented with 1 U/mL of collagenase IV) samples.

### 2.6. Elasticity of ASC–Integra^®^DRT Constructs

Freshly prepared ASC–Integra^®^DRT constructs were cultured up to 2, 7, and 14 days. Then, constructs from each time-point were placed onto a pre-warmed plate (Physica MCR 302, Anton Paar, Graz, Austria) rheometer. The storage modulus was measured with the following parameters: 25 °C, oscillation strain 5%, angular frequency from 0.1 to 600 rad/s at 19 steps, and gap interval: 0.8 mm.

### 2.7. Real-Time PCR

Relative transcript levels in ASCs were normalized to human skin fibroblasts. ASCs and HSFs cultured in the Integra^®^DRT matrix were measured with real-time quantitative polymerase chain reaction (RT-qPCR) 3 weeks after cells seeding. The matrix fragments of equal size (6 mm in diameter) were minced using a spatula and incubated with TRIzol™ Reagent (Thermo Fisher Scientific, Waltham, MA, USA) for 5 min at room temperature. The total RNA was extracted using the TRIzol Plus RNA Purification Kit (Thermo Fisher Scientific, Waltham, MA, USA) according to the manufacturer’s instructions. RNA concentration was measured using NanoDrop ND-1000 instrument (Thermo Fisher Scientific, Waltham, MA, USA). We used 200 ng of the isolated RNA for cDNA synthesis with the High Capacity cDNA Reverse Transcription Kit (Applied Biosystems, Foster City, CA, USA) in a total volume of 20 µL. The reaction was performed in a C1000 Touch Thermal Cycler (Bio-Rad, Berkeley, CA, USA) following the vendor’s recommendations. The obtained cDNA was then mixed with SYBR Green reagent (Applied Biosystems, Foster City, CA, USA) and specific primer pairs (Genomed, Warsaw, Poland) at a concentration of 500 nM. Primer sequences are listed in Table 1. The reactions were run in duplicates on a 7500 Fast Real-Time PCR instrument (Applied Biosystems, Foster City, CA, USA). Relative gene expression levels were calculated based on the ΔCt method using β-2-microglobulin (B2M) as the endogenous control.

### 2.8. Clinical Validation

#### Patient

A 25-year-old woman with severe post-burn contracture as a result of an accident suffered at the age of 10 years was qualified for a surgical treatment. Primarily, she suffered from a flame burn that affected approximately 75% of the total body surface area (TBSA). Around 65% of the TBSA was assessed as a deep, full thickness third-degree burn that required excision to the fascia and closure of wounds by meshed split thickness skin graft. The overall process of healing was severe and was prolonged by local infections. Besides the meshed skin grafts, autologous, cultured, and natural human keratinocytes (NHK) had been applied. The patient was followed up for several years after treatment. Despite the treatment of scars with compression garments, she had developed many contractures during growth and undergone a range of corrective operations as a child. After a few years’ break in the follow-up, the patient visited a plastic surgery clinic for a control examination. Being an adult woman, she presented with severe post-burn contractures and disfigurements and was qualified for a surgical experimental treatment with Integra^®^DRT seeded with autologous ASCs and consecutive compression therapy for prevention of scars.

### 2.9. Advanced Therapy Medicinal Products for Experimental Treatment

ASCs were prepared under Hospital Exemption - Advanced Therapy Medicinal Product (HE-ATMP) under the Good Manufacturing Practices in the Cell Bank at the Faculty of Biochemistry, Biophysics and Biotechnology, Jagiellonian University Gronostajowa 7 St., 30-387 Krakow, Poland.

The procedures of harvesting, isolation, and expansion of ASCs were performed as described in Section 2.1, but in the Good Manufacturing Practice Cell Bank. Finally, 1 × 10^6^ ASC were suspended in 1 mL of 2:1 5% glucose.

#### 2.9.1. Treatment

The patient was qualified to surgical contracture release of the neck (reference place) and right arm combined with Integra^®^DRT reconstruction. The area of the shoulder in the proximity of the right axilla was chosen for the experimental treatment by implantation of Integra^®^DRT seeded with autologous ASCs.

(1) Approximately 3 mL of subcutaneous adipose tissue was harvested by surgical excision.
(2) Three weeks later, scars were excised, and the wound bed was covered with Integra^®^DRT (reference area) or Integra^®^DRT with ASCs (test area) (Figure 5A). During the ongoing excision, the cells were seeded by syringe onto a 10 cm^2^ fragment of Integra^®^DRT at a density of 1 × 10^4^/cm^2^ (Figure 5B). After 15 min of incubation at room temperature, the constructs were placed on the wound bed. Implants were fixed by skin staplers and dressing was applied (Figure 5C).
(3) Four weeks after scar excision, the protective layers from reference and tested places were removed. Integra^®^DRT was covered by the split-thickness skin grafts and NHK suspension. Finally, the post-operative compression therapy was applied.

#### 2.9.2. Follow-Up

The visual assessment of the reconstructed areas was followed up 1, 2, 3, 6, 9, and 12 months after.

### 2.10. Ethical Approval

Tissue samples were obtained in accordance with the positive opinion of the Jagiellonian University—Medical Collage Bioethical Board (1072.6120.10.2017). An experimental treatment was performed according to the procedures approved by the local Bioethics Committee (no. 7/KBL/OIL/2017) with implementation of the recommendations contained in the Declaration of Helsinki. The patient was informed about the risks associated with the experimental treatment and signed an informed consent. The procedure was performed in 2017.

### 2.11. Statistics

Data were processed using OriginPro 2019b (OriginLab Corporation, Northampton, MA, USA). Student’s *t*-test was used to assess the statistical significance of the observed differences.

## 3. Results

### 3.1. Integra^®^DRT Facilitates Growth of Adipose-Derived Stromal Cells

**Figure 1:**
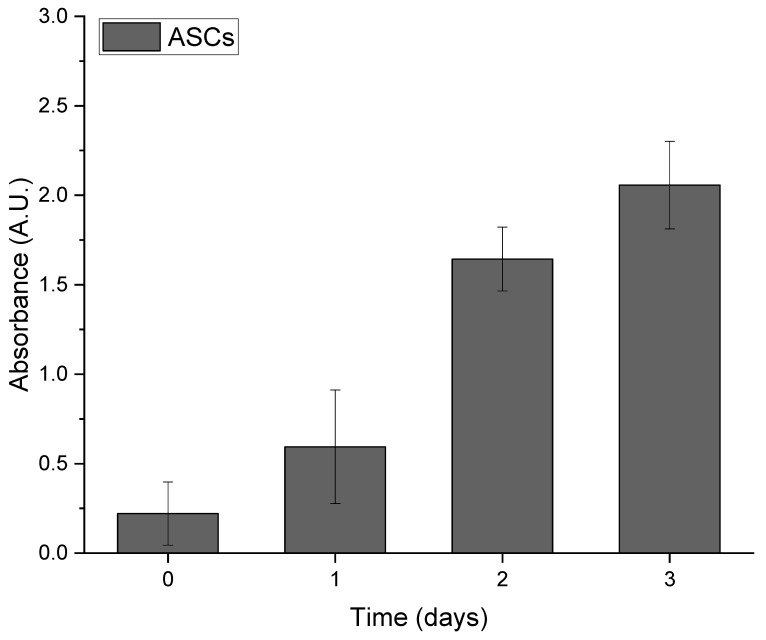
Growth of adipose-derived stromal cells (ASCs) seeded on Integra^®^DRT over long-term observation.

For repetitive viability assessments of ASCs over three weeks, the WST-1 assay was performed at zero, two, and three weeks after cells seeding. The analysis of ASC growth on Integra^®^DRT revealed gradual expansion over the three weeks (Figure 1). The relatively quick clearance of WST-1 allows for repetitive measurements in the same cell–Integra constructs and indicates the typical growth of mesenchymal-derived cells on the collagenous biomaterials.

### 3.2. Proangiogenic and Remodeling Factors Produced by ASCs on Integra^®^DRT

**Figure 2:**
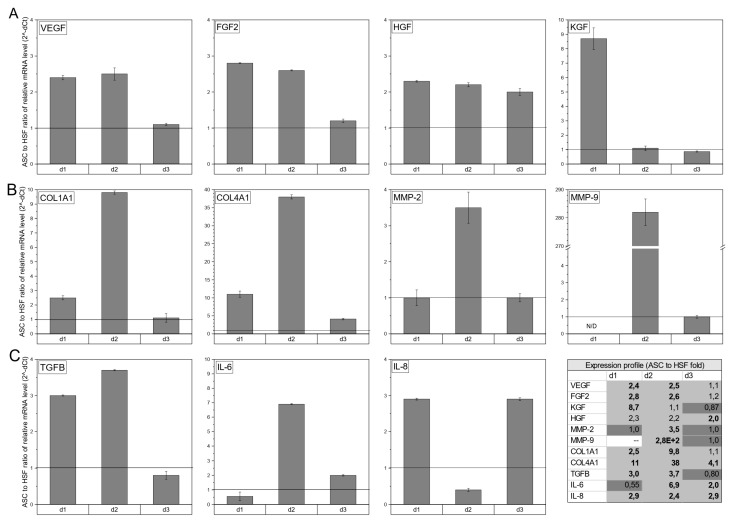
The expression of healing-promoting cytokines (VEGF, FGF2, HGF, and KGF) (**A**), selected tissue-remodeling-associated factors (Collagen I and IV, metalloproteinase (MMP)-2, and MMP-9) (**B**), and immunomodulatory cytokines (TGFβ, IL-6, and IL-8) (**C**). ASCs in compared to human skin fibroblasts (HSFs), both cultured for 3 weeks on Integra^®^DRT matrix. HSFs were harvested from the same donor as ASCs and were used as reference. The transcription profiles of the selected genes are presented as a fold change of relative mRNA levels in ASCs to relative mRNA level in HSFs. The black horizontal line is placed at fold change 1.0. Legend: d1, donor1; d2, donor 2; d3, donor 3.

RT-qPCR was performed to assess the expression of healing-promoting cytokines (VEGF, fibroblast growth factor 2 (FGF2), hepatocyte growth factor (HGF), and keratinocytes growth factor (KGF)), tissue remodeling factors (Collagen I and IV, MMP-2, and MMP-9), and immunomodulatory cytokines (transforming growth factor (TGFβ), IL-6, and IL-8) expressed by ASCs or HSFs cultured on Integra^®^DRT. Among the healing-promoting cytokines (Figure 2A), ASC-based constructs presented higher expressions of VEGF for donor one (2.4×, *p* < 0.05), donor two (2.5×, *p* < 0.05), and donor three (1.1×, non-significant); FGF2 for donor one (2.8×, *p* < 0.05), donor two (2.6×, *p* < 0.05), and donor three (1.2×, non-significant); HGF for donor one (2.3×, non-significant), donor two (2.2×, non-significant), and donor three (2.0, *p* < 0.05); and KGF for donor one (8.7×, *p* < 0.05) and donor two (1.1×, non-significant) in comparison with HSF-based Integra^®^DRT. The tissue-remodeling-associated transcripts in ASC vs. HSF constructs (Figure 2B) were expressed at a higher level for Col1A1 for donor one (2.5×, *p* < 0.05), donor two (9.8×, *p* < 0.05), and donor three (1.1×, non-significant); Col4A1 for donor one (11×, *p* < 0.05), donor two (38×, *p* < 0.05), and donor three (4.1×, *p* < 0.05); MMP-2 for donor two (3.5×, *p* < 0.05); and MMP-9 for donor two (280×, *p* < 0.05).

The immunomodulatory expression profile (Figure 2C) revealed a higher relative mRNA level in ASC-based compared to HSF-based Integra^®^DRT constructs of TGFβ for donor one (3.0×, *p* < 0.05) and donor two (3.7×, *p* < 0.05); IL-6 for donor two (6.9×, *p* < 0.05) and donor three (2.0×, *p* < 0.05); and IL-8 for donor one (2.9×, *p* < 0.05), donor two (2.4×, *p* < 0.05), and donor three (2.9×, *p* < 0.05). qPCR revealed that compared to HSFs, ASCs presented a highly healing-promoting profile that was not strongly correlated with the donor’s cells.

### 3.3. ASCs Spread among the Integra^®^DRT

**Figure 3:**
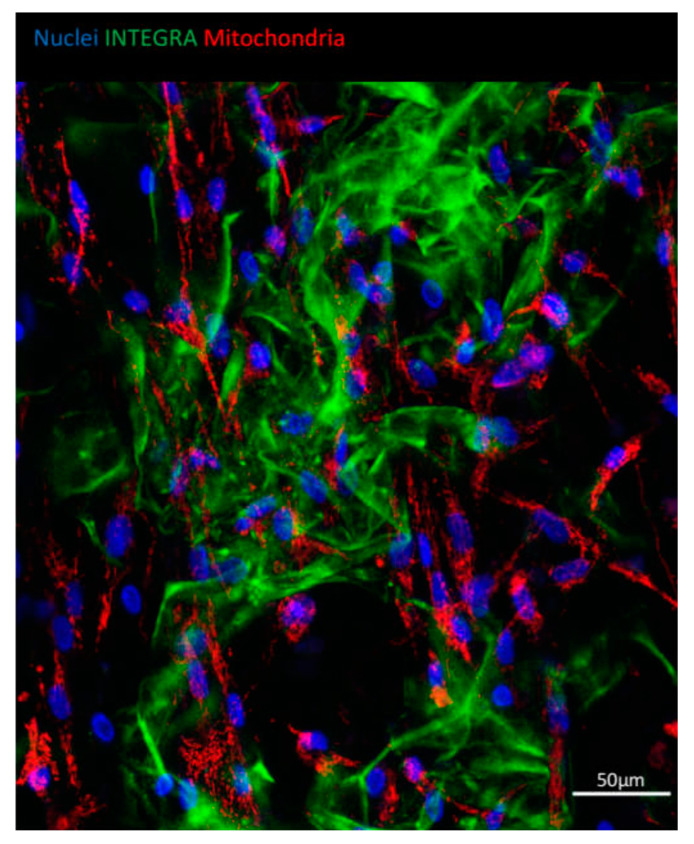
ASCs growing on Integra^®^DRT. Viability of cells evidenced by a mitochondrial high membrane potential of ASCs shown with tetramethylrhodamine, ethyl ester (TMRE) staining (red). Nuclei were counterstained with Hoechst 33342 (blue); autofluorescence of Integra^®^DRT was visualized in the green channel.

To visualize the ASCs growth on Integra^®^DRT, nuclei and mitochondria were stained with Hoechst 33342 and TMRE, respectively, at the third week post-cell-seeding. Then, fluorescence confocal scanning microscopy was performed to measure blue and red fluorescence emission. Figure 3 reveals that ASCs attached to Integra^®^DRT as fibroblast-like cells. Each adipose-derived stromal cell presented viable mitochondria observed as red-fluorescent perinuclear structures (Figure 3). Autofluorescence of collagen-based Integra^®^DRT was measured in the green channel.

### 3.4. Decrease in ASC-Integra^®^DRT Elasticity Correlates with Collagenolytic Activity

**Figure 4:**
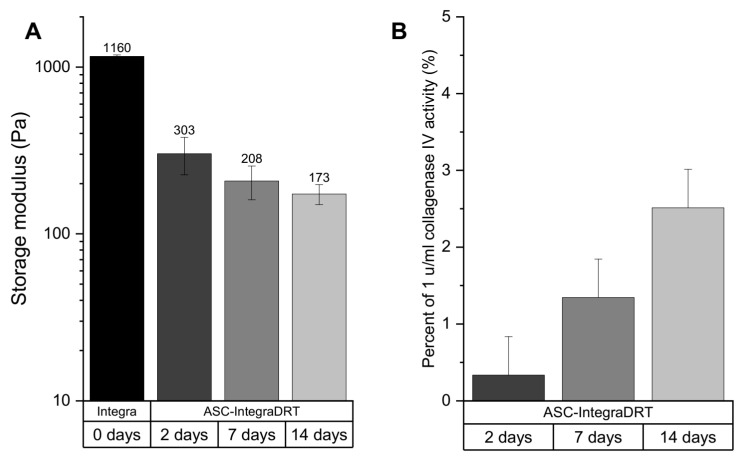
The elasticity of ASC–Integra^®^DRT constructs on days 2, 7, and 14 after cell seeding expressed as storage modulus (**A**). Collagenolytic activity of the constructs (**B**). The decrease in ASC–Integra^®^DRT elasticity was positively correlated with the increase in the proteolytic activity at the same time points.

The storage modulus of Integra^®^DRT incubated in the cell culture media decreased over time by seeded ASCs (Figure 4A). The initial Integra^®^DRT storage modulus was 1.1 kPa and decreased in ASC–Integra^®^DRT constructs to 303 Pa on day 2, 208 Pa on day 7, and 172 Pa on day 14. Elasticity of Integra^®^DRT is expressed as storage modulus, which depends on the network density of the biomaterial (collagen and sulfated glycosaminoglycans) [22]. For this reason, the collagenase activity of the constructs at the same time points were checked, which revealed an increasing collagenolytic activity over time. Data were normalized to negative and positive controls as a percentage of 1 U/mL collagenase IV (this activity is sufficient to complete digestion of Integra^®^DRT overnight). On the second day of ASCs culturing on Integra^®^DRT, we detected 0.3% of the normalized activity, which increased to 1.3% and 2.5% on days 7 and 14, respectively (Figure 4B).

### 3.5. ASC Cellular Therapy Reveals Anti-Fibrotic Healing

**Figure 5:**
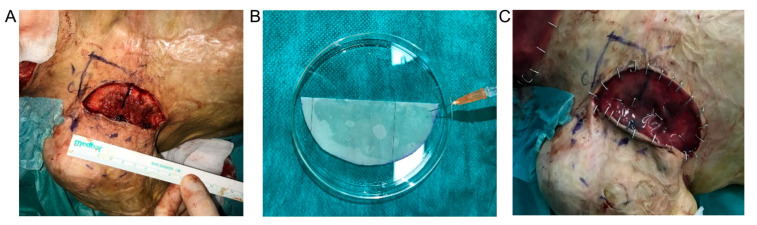
The second surgery: Scar excision from the tested area (**A**), ASCs seeding onto Integra^®^DRT (**B**), and reconstruction of the tested area with Integra^®^DRT coated by ASCs after skin staplers fixing (**C**).

**Figure 6:**
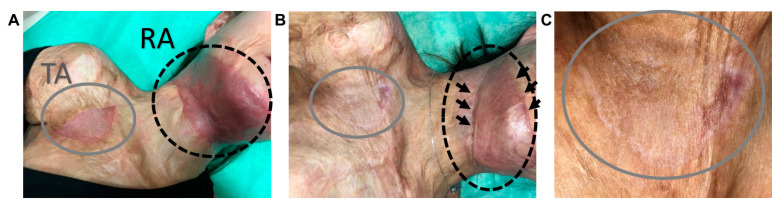
The visual assessment performed during a 4-week follow-up (**A**) and 9 months (**B**,**C**) after the last surgery. A enlargements of the tested area (**C**). TA, the tested area; RA, the reference area.

An approximately 10 cm^2^ post-scar-excision defect at the upper right axilla (Figure 5A) was reconstructed with suitable Integra^®^DRT with seeded autologous ASCs (Figure 5B,C, tested area, TA). The frontal neck area, reconstructed with Integra^®^DRT with no cells, was chosen as the reference area (RA). After four weeks, the tested and the reference area were covered with split-thickness skin grafts and NHK suspension. All three operations were performed with no adverse effects. During a follow-up, the tested area was less reddish and more elastic than the reference area (Figure 6A). Nine months after the last surgery, the border between Integra^®^DRT and the non-reconstructed skin was deprived from the scar in the tested area when compared to the reference area where the circumference presented a visible scar (Figure 6B,C). The tested area became as flesh-colored as the surrounding skin in contrast to the whole distinctly reddish reference area. One year after the intervention, the difference between the tested and the reference area remained but no other undesirable effects were observed (not shown).

## 4. Discussion

In this paper, we presented a new approach for reconstruction of a burn-related scar using a combination of a collagen-based scaffold and autologous adipose-derived stromal cells. Here, we showed that in comparison to skin fibroblasts, both cultured in vitro on Integra^®^DRT, ASCs more efficiently express numerous factors with pro-regenerative functions. As we investigated ASC and HSF transcripts, numerous studies provided evidence of similar expression profiles on the proteomic level [23,24,25]. During physiological sequence of wound healing, VEGF is released by keratinocytes and macrophages to facilitate angiogenesis from the wound bed [26]. This spontaneous process, underlying biomaterial-mediated healing, is modified by a restriction of oxygen diffusion across the biomaterial. The mild hypoxia that occurs inside the biomaterials (approximately 10–15% O_2_ at around a 1000 µm depth) [26] induces expression of hypoxia inducible factor-1 (HIF-1) in ASCs and finally VEGF production. Our findings indicated that ASCs seem to be a more efficient source of growth factors, including VEGF and pro-angiogenic IL-6 compared to skin fibroblasts. This corresponds to the results obtained by Lee et al. [18] who compared ASCs with HSFs in a normo- and hypoxic conditions in a two-dimensional cell culture.

The secretome of ASCs promotes angiogenesis and induces tissue remodeling [27]. ASCs growth on Integra^®^DRT is not affected, and mesenchymal stem cells are able to remodel the microenvironment by collagens I and IV and fibronectin deposition as well as MMP-3 and MMP-9 production. Our data suggested that despite biomaterial enrichment in extracellular matrix (ECM) proteins or the presence of growing cells, ASC-mediated remodeling has no effect on the elasticity of ASC–Integra^®^DRT constructs. A relatively low-intensity mechanical stress (below 1 kPa) that acts on cells inside biomaterials deters fibrosis [15,16]. This physiological process, which occurs during wound healing [28], is overrated by the sustained mechanical stress or chronic inflammation, leading to fibrosis. The increasing collagenolytic activity together with MMP-2 and MMP-9 expression by ASC–Integra^®^DRT constructs showed that ASCs slightly digest the ECM and decrease the mechanical stress. From that point of view, ASCs seeded onto Integra^®^DRT inhibited fibrosis via secretomes [29,30]. Yates et al. [31], referring to the CXCR3-deficient mice model of wound-scaring, revealed that co-transplantation of both mesenchymal stem cells (MSCs) and HSFs improves healing; however, MSCs are responsible for decreasing fibrosis [31]. Taken together, implantation of Integra^®^DRT with ASCs strongly stimulates angiogenesis precisely at the place of biomaterial contact with the wound bed.

The process of Integra^®^DRT remodeling by mesenchymal stem cells is enhanced by monocytes infiltration [32]. Walter et al. [33] indicated that mesenchymal stem cells stimulate host fibroblasts to migrate toward the wound [33]. Another cytokine released by ASCs, IL-6, stimulates proliferation and migration of keratinocytes in the wound, which improve epidermis reconstitution [34]. Our data indicated that ASCs produce strongly chemoattractive agents such as IL-6, IL-8, FGF-2, and TGF-β which, with respect to the data provided by Chen et al. and Walter et al. [32,33], explains the ASCs interaction with host cells. Chen at al. found that the conditioned medium collected from bone marrow mesenchymal stem cells but not from dermal fibroblasts cell culture contains high concentrations of macrophage inflammatory proteins, which result in higher CD4/80 positive monocytes/macrophages content in cytometry analysis of tissue samples. The same study indicated that VEGF and stromal cell-derived factor 1, as well as other factors present in the MSC-conditioned medium, improve wound closure by enhancing vascularization and remodeling.

As vascularization and remodeling seem to be key players for non-scar healing, the rationale for autogenic or allogenic mesenchymal stem cells transplantation is still unclear. Increasing scaffold cellularity by autologous cells transplantation improves healing by MSC differentiation, but MSC-associated healing is also observed in allo- or xenogeneic therapy [31,35]. In our three-week in vitro observation, ASCs remained alive; however, in vivo data suggested that cells are dying after transplantation. Muhammad et al. [35] showed that allogenic ASCs transplantation in acid-related burns of immunocompetent mice strongly accelerates wound closure despite the gradual loss of transplanted ASCs. From that point of view, the transient presence of ASCs or secretome alone seems to be the key player in a soft tissue regeneration.

Instead of numerous attempts to prevent scars, contractures must be managed, particularly when restriction of joint movement occurs [8,10,13]. Scar excision and the following reconstruction of the skin by Integra^®^DRT combined with skin grafts or keratinocytes auto-transplantation is a recognized method of contracture management; regardless, new scars occur along the edges of the patch [5,10,13]. Several in vivo studies provided the first concepts of mesenchymal stem cells transplantation on collagen-based scaffolds in wound management [36,37,38,39,40]. Here, we validated this concept through experimental treatment of human skin reconstruction in burn-related scar excision. Non-scar healing was observed along the border of the graft and host tissue where fibrosis typically occurs. No safety issues were reported. As rapid bench-to-bedside translation was used to combine a clinically approved medical device with HE-ATMP, a legislative formula of the experimental treatment enables the proper design of future clinical trials.

## Figures and Tables

**Table 1:** Primers used for real-time qPCR.

Gene	Forward (5′–3′)	Reverse (5′–3′)
*COL1A1*	AAGAGGAAGGCCAAGTCGAG	CACACGTCTCGGTCATGGTA
*COL4A1*	GGTATTCCAGGATGCAATGG	GCACATGGCCAAGTATCTCA
*VEGF*	TACCTCCACCATGCCAAGT	TGCATTCACATTTGTTGTGC
*KGF*	TGCCAACTTTGCTCTACAG	CACTTTCCACCCCTTTGA
*MMP2*	CGCTACGATGGAGGGGCGCTA	AGAAGGTGTTCCAGGTATTTGCACTG
*MMP9*	CGCAGACATCGTCATCCAGT	AACCGAGTTGGAACCACGAC
*IL-6*	GTGAAAGCAGCAAAGAGGCA	TCACCAGGCAAGTCTCCTCA
*Il-8*	TTAGCACTCCTTGGCAAAACTG	CTGGCCGTGGCTCTCTTG
*HGF*	TCACGAGCATGACATGACTCC	AGCTTACTTGCATCTGGTTCC
*FGF2*	AAAAACGGGGGCTTCTTCCT	TGTAGCTTGATGTGAGGGTCG
*TGF* *β*	GGACATCAACGGGTTCACTAC	TGAGAAGCAGGAAAGGCCG
*B2M*	GATGAGTATGCCTGCCGTGTG	CAATCCAAATGCGGCATCT
